# Sleep and cardiovascular disease

**DOI:** 10.1042/ETLS20230111

**Published:** 2023-12-12

**Authors:** Michelle A. Miller, Nathan E. Howarth

**Affiliations:** Division of Health Sciences (Mental Health and Wellbeing), Warwick Medical School, University of Warwick, Gibbet Hill, Coventry CV4 7AL, U.K.

**Keywords:** cardiovascular disease, inflammation, obesity, risk markers, sleep disorders

## Abstract

This review centres around the recent evidence in examining the intersection of sleep and cardiovascular disease (CVD). Sleep in this review will be further subdivided to consider both sleep quantity and quality along and will also consider some of the more common sleep disorders, such as insomnia and obstructive sleep apnoea, in the context of CVD. Sleep disorders have been further explored in several specific populations which are both at risk of sleep disorders and CVD. Secondly, the review will present some of the risk factors for CVD that are affected by sleep and sleep disorders which include hypertension, diabetes, and obesity. It will also examine the potential underlying mechanisms including inflammation, appetite control, endocrine, and genetic processes that are affected by sleep and sleep disorders leading to increased risk of CVD development. In addition, we will consider the observed bi-directional relationships between sleep and cardiovascular risk factors. For example, obesity, a risk factor for CVD can be affected by sleep, but in turn can increase the risk of certain sleep disorder development which disrupts sleep, leading to further risk of obesity development and increased CVD risk. Finally, the review will explore emerging evidence around lifestyle interventions that have included a sleep component and how it impacts the management of CVD risk factor. The need for increased awareness of the health effects of poor sleep and sleep disorders will be discussed alongside the need for policy intervention to improve sleep to facilitate better health and well-being.

## Background

Quantity and quality of sleep are affected by many different parameters including both personal and environmental factors. A reduction in the average hours of sleep across westernised populations has been reported, which may in part reflect changes in societal working hours and increasing shift work, with increasing reports of fatigue, tiredness, and excessive daytime sleepiness (EDS). In the U.S.A., the percentage of adults sleeping 6 h or less a night increased by 31% sleep duration has reduced amongst US adults in the period from 1985 to 2012 [1]. Short sleep is associated with an increased risk of multiple adverse health outcomes including total mortality [2] and this review will examine evidence that suggests that both poor sleep (quantity and quality) and sleep disorders are independently associated with increased risk of cardiovascular disease (CVD).

## Sleep

Poor or unhealthy sleep might reflect either a difficult in initiating sleep, difficulty maintaining sleep (DMS), or waking up tired. It may arise because of an individual's inability to obtain sufficient sleep or sleep of sufficient quality. This may result from an individual's lifestyle choices, work demands, environmental factors, or be due to underlying medical conditions that cause sleep disturbances. Ideally, it is suggested that an adult should obtain 7–9 h of sleep per night [3] that is maintained at a regular time each day, during normal nighttime hours and, should be uninterrupted.

## Sleep disorders

There are over 70 known sleep disorders many of which are associated with poor sleep including insomnia, obstructive sleep apnoea (OSA), and periodic limb movement disorder (PLMD).

Insomnia is characterised by a feeling of difficulty initiating sleep (DIS), DMS, waking up early or waking up feeling tired (non-restorative sleep (NRS)), and daytime sleepiness. OSA is a chronic, sleep-related breathing disorder, which is associated with sleep fragmentation. Insomnia and OSA are two of the most common sleep disorders which can cause EDS, that is an increased propensity to fall asleep during the day, even in inappropriate circumstances.

PLMD is a condition in which people experience repetitive periodic limb movements (PLMS) such as jerking, cramping, or twitching in their lower limbs during sleep, and which can cause sleep disturbances.

## Sleep and cardiovascular disease

CVD can be subdivided into different types including coronary heart disease (CHD), stroke, peripheral artery disease, and aortic disease. Significant relationships have been demonstrated between short sleep and both CHD and stroke [4]. Chandola et al. [5] demonstrated that that cardiovascular mortality was mainly greater in short sleepers who also experience poor sleep quality.

**Figure 1.**
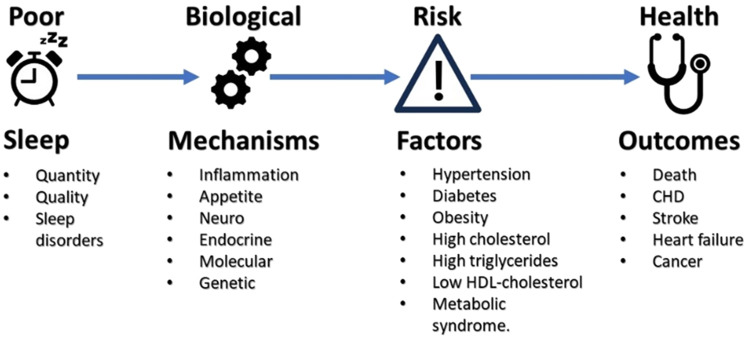
Relationship between adverse health outcomes and sleep: Risk factors and underlying mechanisms.

Poor sleep may have many biological effects on metabolic, endocrine, and immune systems [6] and adversely affect risk factors associated with cardiovascular risk (see Figure 1). The relationship, however, appears to be complex with both short and long sleep associated with many adverse health outcomes [7,8]. A recent meta-analysis suggested that both long sleep duration and poor sleep quality were associated with arterial stiffness (AS), a key risk factor for hypertension and CVD [9]. In this review, we will outline some of the plausible mechanisms by which short sleep may lead to an increase in CVD, including inflammatory, appetite, endocrine, and genetic mechanisms. To date, however, there are no studies that have demonstrated a possible mechanisms for the association between long sleep and CVD. This suggests that different underlying mechanisms/causal pathways may be important for the associations seen for long and short sleep. Indeed, it has been suggested that the association between long sleep and CVD may be explained by residual confounding and co-morbidities. Factors that might be important include depressive symptoms, socio-economic and employment status, and the presence of yet undiagnosed health conditions.

## Sleep disorders and CVD risk

### Insomnia and CVD risk

In women compared with men, the prevalence of insomnia may be higher, with an overall prevalence in the general population of 22.0% [10]. Wu et al. [11] demonstrated that insomnia was associated with an increased summary relative risk (SRR) of atrial fibrillation (AF) (SRR: 1.30, 95% CI: 1.26–1.35), cardiovascular diseases (1.45, 1.29–1.64), CHD (1.28, 1.10–1.50), myocardial infarction (MI) (1.42, 1.17–1.72), and stroke (1.55, 1.39–1.72). Insomnia and sleep duration of less than 5 h per night has also been shown to be highly associated with an increased incidence of MI, with a difficulty in initiating or maintaining sleep but not with NRS of daytime dysfunction [12].

### Obstructive sleep apnoea and CVD risk

OSA is characterised by repetitive partial or complete blockages of the airway during sleep, leading to interruptions in breathing, blood oxygen desaturation, and arousal from sleep which may cause chronic sleep deprivation. It is associated with an increased CVD risk, acting through inflammation, neurohormonal dysregulation, and endothelial dysfunction [13]. Estimates suggest that in western countries at least 10% of females and 20% of males are affected by asymptomatic OSA [14]. In some patients OSA is associated with EDS, these patients are classified as having obstructive sleep apnoea syndrome (OSAS) [15]. The British Lung Foundation suggests that many with OSA/OSAS remain undiagnosed [16]. World Health Organization (WHO) estimates from 2007 suggested that 100 million people around the world were affected by OSA. Much higher prevalences were reported in a more recent study by Benjafield et al. [17]; estimating the global prevalence of OSA in individuals 30–69 years old that may have mild-to-serve OSA at 936 million individuals, of these 425 million may have moderate-to-severe OSA. If untreated, it leads to poor health outcomes [18–20] and has been shown to be a risk factor for Arrhythmias, Conduction Disorders, and Cardiac Arrest [21]. A recent meta-analysis demonstrated that OSA was associated with a 74% relative increased risk of all-cause cardiac death and 94% increased risk of cardiovascular mortality [22].

OSA is a known risk factor for hypertension in adults and a recently significantly higher mean systolic BP (SBP) was observed in children with mild or moderate-to-severe OSA compared with healthy controls. Furthermore, in the prospective studies, moderate-to-severe childhood OSA was associated with a risk of elevated SBP in adulthood. Early detection and treatment of OSA may promote cardiovascular health in children and possibly in future adulthood [23].

Obesity is a risk factor for the development of OSA, with increased tongue size and fatty deposits in the neck which occlude the airway. This in turn can lead to fragmented sleep which may lead to metabolic and hormonal changes that increase appetite and lead to further weight gain thus potentiating a vicious bi-directional pathway of increased risk of obesity and increased severity of OSA and CVD [24,25].

The intermittent drops in oxygen saturation (hypoxia), abrupt drops in intrathoracic pressure, sympathetic activation, and inflammatory disturbances associated with OSA can cause acute and long-term adverse implication for heart and blood vessels. A recent study indicates that right-ventricular free-wall and global longitudinal strain are impaired in patients with OSA. Moreover, the deterioration of these indices was evident in the early stages of the disease and was related to disease severity [26]. Individuals with OSA suffer from oxidative stress because of the hypoxemic episodes associated with this condition. These in turn can induce a type of arrhythmia known as AF. A recent meta-analysis of sixteen observation studies showed that the risk of AF increased with increasing OSA severity as determined by measuring the apnea–hypopnea index (AHI) in a dose-dependent manner [27].

OSA has also been shown to be associated with an increased risk of cerebral vessel disease, a major contributor to adverse health outcomes, including stroke; moderate-severe OSA was associated with a higher risk of lacunar infarcts, but no association with cerebral microbleeds [28]. In acute coronary syndrome patients OSA is associated with a significant increase in the risk of cardiovascular events [29].

### OSA in other population groups

In certain rare diseases, the prevalence of OSA is markedly higher than that among the average population, such as in Ehlers–Danlos syndrome, a connective tissue disorder [30] and Marfan syndrome in which the upper airway is excessively collapsible [31,32]. Both conditions demonstrate excess CVD risk [33].

Polycystic ovary syndrome (PCOS) disease is a prevalent disorder affecting 8–13% of reproductive-age women [34]. In a recent systematic review and meta-analysis, PCOS participants had a 6.22-fold risk (OR) of sleep disturbance, which included sleep-disturbed breathing and OSA, compared with non-PCOS participants [35,36].

It has also been reported to be higher in other specific populations including sportsmen such as American football players; despite being athletes, some evidence suggests the size of the players, particularly lineman, is associated with a high prevalence of obesity which might have contributed to their increased susceptibility to develop OSA [37,38]. Sleep disorders are also prevalent in professional rugby players [39,40] and athletes undertaking contact sports have higher prevalence of OSA which may be the result of a head injuries [41].

Shift work is associated with an increased risk of CVD with the risk of any CVD event being 17% higher among shift workers than day workers [42]. The risk of CHD morbidity was 26% higher. Furthermore, after the first 5 years of shift work, there was a 7.1% increase in the risk of CVD events for every additional 5 years of exposure.

In a recent meta-analysis, the prevalence of sleep disorders in first responders was found to be, 31% for shift work disorder (SWD), 30% for OSA, 28% for insomnia, 28% for EDS, 2% for restless leg syndrome, and 1% for narcolepsy. First responders with OSA were also more likely to be at risk of developing CVD alongside anxiety, depression, diabetes, gastroesophageal reflux disease, and post-traumatic stress disorder compared with those without [43].

### COMISA (Co-Morbid Insomnia and Sleep Apnoea)

In some individuals, co-morbid insomnia and OSA co-occur as COMISA. These individuals have higher rates of hypertension and CVD at baseline, and an increased risk of all-cause mortality compared with no insomnia/OSA [44].

### Somnipathy, diabetes, and CVD risk

Both abnormal or disordered sleep ‘somnipathy’ and diabetes have been shown to be associated with an increased risk of CVD. A study evaluated the risk of the coexistence of somnipathy (which includes insomnia and sleep-related breathing disorders, central disorders of hypersomnolence, circadian rhythm sleep–wake disorders, sleep-related movement disorders, parasomnias, and other sleep disorders) and diabetes. It showed that the presence of both was associated with higher risks of CVD, CHD, stroke, and mortality than when an individual had either somnipathy or diabetes alone [45].

## Risk factors for CVD and association with sleep and sleep disorders

The suggested link between poor sleep and risk factors for CVD and the potential underlying mechanisms is shown in Figure 1. Each of these risk factors and potential mechanism will be briefly considered below.

### Hypertension

A lack of sleep is associated with an increased risk of hypertension [46,47]. PLMS is also associated with an increased risk of hypertension in cross-sectional studies, but further prospective studies would be required to demonstrate causality [48].

### Diabetes

Early meta-analyses of prospective studies support an epidemiological link between quantity (short and long duration) and quality of sleep (difficulties in initiating or in maintaining sleep) and the subsequent development of type-2 diabetes (T2DM) [49]. In a recent meta-analyses, a potential dose–response relationship was observed between the severity of the (OSA) and the risk of T2DM [50]. In a separate study, OSA was associated with a higher risk of impaired fasting glucose, impaired glucose tolerance, impaired glucose regulation, and diabetes mellitus in both cohort and cross-sectional studies; moreover, the severity of diabetes increased with the severity of OSA [51].

### Obesity

An elevated body mass index (BMI) is a major risk factor for heart disease, stroke, T2DM, and other chronic diseases including OSA. Overweight individuals are defined as having a BMI of 25–30 kg/m^2^, and obese individuals having a BMI > 30 kg/m^2^. Increasing evidence from cross-sectional [52] and more recent prospective studies, in which short sleep proceeds the subsequent weight gain in infants and children [53–55], support a link between short sleep and the development of obesity. This suggests that the relationship is causally related and may contribute to obesity development, but it is possible that there may be many different underlying mechanisms.

## Mechanisms

### Inflammation

Sleep deprivation is associated with markers of inflammation including interleukin 6 and C-reactive protein (CRP) [6,56–58], which are associated with increased risk of CVD. Usually during sleep, blood pressure decreases, and blood vessels relax; however, it has been proposed that if sleep is restricted and blood pressure remains elevated there may be an effect on the blood vessel that leads to an increase in inflammation. Several inflammatory mechanisms, associated with the pathophysiology and progression of OSA, have also been associated with COVID-19 disease. This may increase the risk of poor COVID-related outcomes in individuals with OSA [59]. A lack of sleep has also been associated with higher circulating lipids [60] and the development of obesity which might also lead to the activation of inflammatory pathways [61].

### Appetite

A lack of sleep may influence various hormonal responses affecting both hunger, satiety, and appetite control [62] which would increase appetite. Spiegel et al. [63], in a randomised cross-over trial, demonstrated that acute sleep deprivation was associated with a decrease in the satiety hormone leptin and an increase in the hunger hormone ghrelin and, despite a glucose infusion to maintain caloric intake, an increase in hunger. Likewise in the Wisconsin Sleep Cohort Study individuals who slept less than 8 h had an increased in BMI and lower leptin and higher ghrelin levels [62]. Serum leptin levels have also been shown to be elevated in children with OSA and correlated with BMI [64].

### Endocrine

Sleep restriction is associated with reduced insulin sensitivity, and circadian misalignment and slow-wave sleep suppression negatively affect insulin sensitivity [65]. This in turn may lead to high glucose levels and diabetes risk which over time leads to damage to the arteries and subsequent build-up of fatty material within them leading to the development of atherosclerosis and CVD. The renin–angiotensin–aldosterone system (RAAS) is important for regulating salt and water homeostasis and blood pressure control as well as cardiovascular remodelling. It was recently found that patients with OSA have higher levels of RAAS hormones, blood pressure, and heart rate compared with those without OSA [66].

### Genetic

Many biological processes within the body run on an approximately 24-h cycle that is controlled by the action of light on the master clock located in the suprachiasmatic nuclei in the brain. This in turn regulates clocks in other body tissues and is influenced by the action of many genes including the ‘clock’ genes Per, tim, and Cry. These genes govern the timing of many physiological processes such as the 24 h variation in glucose levels [67]. Studies in mice suggest that genetic mutations of these genes can affect metabolism and lipid and glucose metabolism [68]. These processes, however, can be disrupted when individuals sleep outside the normal light–dark cycle for example in shift work and this can lead to alternations in metabolism and increased CVD risk [69].

In a recent Mendelian randomisation study, it was demonstrated that there is evidence to suggest casual evidence for both unidirectional and bi-directional relationships between sleep and adiposity [70].

Genes also determine an individual's chronotype, which determines whether they are more likely to have a natural inclination to wake up early and go to bed early (lark) or to get up later and go to bed later (owl). A longer allele on the PER3 circadian clock gene is associated with being a lark [71]. Evening chronotype individuals may have a higher risk of obesity and a worse metabolic profile [72]. A recent study conducted in teenagers, suggested that this may be due to differences in food preferences [73]. They found that teenagers who reported later sleep timing were more likely to consume sugary/caffeinated beverages and high-energy-dense, nutrient-poor foods.

Eating patterns are affected by a lack of sleep. In one study short sleeping individuals had an increased intake of snacks and fatty and sweet foods but decreased intake of fruit and vegetables [74].

A recent genome-wide association study (GWAS) meta-analysis of sleep apnoea uncovered multiple genetic loci associated with the sleep apnoea. These included five independent significant loci associated signals that spanned chromosomes 5, 11, 12, and 16 near genes ANKRD31, STK33, BDNF, KDM2B, and PRIM1. Whilst adjustment for BMI as a covariate led to a significant reduction in the strength of these associations it also identified a new significant locus on chromosome 15 near HDGFL3 and one on chromosome 13 near DLEU1 and DLEU7. This study observed genetic correlations with several complex traits, including multisite chronic pain, diabetes, high blood pressure, chronic obstructive pulmonary disease, and BMI-associated conditions. Findings from their study also suggested that the levels of sex hormone-binding globulin (SHBG) would be predicted to reduce the risk of OSA [75].

A separate study, which utilised samples from the UK biobank, concluded that there was evidence for pathway-specific genetic risk factors of coronary artery disease (CAD) that differ between individuals with and without OSA in a qualitatively pathway-dependent manner [76].

### Social factors

In pre-school-aged children, it has been shown that regularly eating the evening meal as a family, obtaining adequate nighttime sleep, and having limited screen-viewing time were associated with lower prevalence of obesity compared with those children who did not have these routines [77]. An earlier bedtime in pre-school children has also been shown to be associated with a lower BMI scores and lower intake of added sugars [78].

## Lifestyle intervention programmes and treatments

### Sleep and weight loss

Results from a randomised cross-over trial in healthy adults suggest that insufficient sleep may determine what proportion of fat to muscle is loss on a calorie-controlled diet [79]. The investigators found that when individuals who were either overweight or obese were assigned to the 8.5 h sleep period, they lost most of their weight as fat mass but when they were assigned to the 5 h sleep period they loss more of the weight as muscle mass. These individuals also reported an increase in appetite when they slept only 5.5 h, which might affect an individual's ability to lose metabolically active fat mass on a calorie-controlled diet.

### Sleep extension studies in children

In a systematic review and meta-analysis of five intervention studies that aimed to improve sleep in pre-school children, it was found that there was a beneficial effect on BMI [80]. There was, however, a significant degree of heterogeneity between the different interventions used and further studies are required to investigate this further.

### Sleep extension studies in adults

Sleep extension may have beneficial metabolic effects and may be a useful adjunct therapy for weight management [81]. Al Khatib et al. [82] assessed the feasibility of a personalised sleep extension protocol in adults aged 18–64 years who were habitually short sleepers (5 to <7 h). Individuals who received targeted sleep hygiene significantly increased their time in bed and sleep duration. Furthermore, the sleep extension group reduced their intake of fat, carbohydrates, and free sugars as compared with the control group.

### Napping

There has been some debate as to whether napping is of benefit to health and whilst various studies have found that short daytime naps (10–30 min) can increase performance and make you more productive at work it is possible that longer naps might be the result of a ‘need to sleep’ due to an underlying pathology i.e. EDS or OSA. A recent study suggested napping was associated with an increase in brain size, which might have a beneficial effect of preserving memory and cognitive function in older adults [83]. But, in a British cohort study of over 16 000 men and women, it was shown that daytime napping was associated with and increased risk of all-cause mortality [84]. A recent genetic mendelian randomisation study also demonstrated that more frequent daytime napping was significantly associated with higher odds of coronary atherosclerosis, MI, and heart failure. Regular napping during the daytime was also associated with increased of CVD primarily through the development of atherosclerosis [85]. Furthermore, in a dose–response meta-analyses it was shown that daytime napping <30 min/d was not significantly associated with higher odds of most CVD risk factors and CVD among young and middle-aged adults but in older adults aged >60 years, a significant dose–response association of any daytime napping with higher odds of diabetes, dyslipidemia, MetS, and mortality was observed [86].

## Treatment for OSA and CVD risk

OSA is associated with the CVD, which frequently requires continuous positive airway pressure (CPAP) to keep the airway open at night. Marin et al. [87] have shown a beneficial effect of CPAP on CVD risk in individuals with severe OSA. Although a recent randomised control trial failed to demonstrate a statistically significant reduction in the incidence of cardiovascular events in patients with OSA treated with CPAP^.^ [88]. The reasons for these differences are unclear, but the authors do cite several limitations to their study and suggest that the study may have been inadequately powered in relation to the sample size. CPAP treatment does, however, have a beneficial effect on hypertension [89] and cardiovascular autonomic function [90].

Inflammatory markers including CRP are increased in OSA [91] and inflammatory (CRP, IL-6, and TNF-α) and cardiometabolic profiles (total cholesterol, LDL, triglyceride) as well as leptin are improved following soft-tissue surgery for OSA [92]. Likewise, the neutrophil-to-lymphocyte ratio (NLR), a measure of subclinical systemic inflammation, is significantly reduced in patients using CPAP [93].

## Policy intervention

Sleep is affected by several factors including an individual's physical and mental health but also by environmental (such as light and noise pollution) and social factors (such as shift work). Short-term disruptions can affect performance and lead to a risk of accidents, but long-term disturbances can lead to poor health and increased CVD risk. Public health strategies are needed to increase the awareness of the importance of healthy sleep habits and to improve the diagnosis and treatment of sleep disorders [94].

## Summary

- Chronic sleep loss and poor-quality sleep are risk factors for CVD development.
- Sleep disorders are often undetected, yet the prevalence may be very high in specific populations, including athletes, and warrants further investigation.
- Detection and treatment of poor sleep and sleep disorders are important for CVD prevention in both adults and children.
- Improving sleep may aid weight regulation and management in children and adults and reduce CVD risk.
- There is a need for policy intervention to improve sleep to facilitate better health and well-being.

## Abbreviations

AF: atrial fibrillation
BMI: body mass index
CHD: coronary heart disease
CPAP: continuous positive airway pressure
CRP: C-reactive protein
CVD: cardiovascular disease
DMS: difficulty maintaining sleep
EDS: excessive daytime sleepiness
MI: myocardial infarction
NRS: non-restorative sleep
OSA: obstructive sleep apnoea
OSAS: obstructive sleep apnoea syndrome
PCOS: polycystic ovary syndrome
PLMS: periodic limb movements
RAAS: renin–angiotensin–aldosterone systems
SBP: systolic BP
SRR: summary relative risk
